# Exploring the incidence and risk factors of reoperation for symptomatic adjacent segment disease following cervical decompression and fusion

**DOI:** 10.1016/j.xnsj.2023.100305

**Published:** 2023-12-22

**Authors:** Hania Shahzad, Paul M. Alvarez, Mustaqueem Pallumeera, Nazihah Bhatti, Elizabeth Yu, Frank M. Phillips, Safdar N. Khan, Varun K. Singh

**Affiliations:** a Department of Orthopedics, The Ohio State University, Wexner Medical Center, 241 W 11th Ave, Suite 6081, Columbus, OH 43201, USA; b Ohio State University, 241 W 11th Ave, Suite 6081, Columbus, OH 43201, USA; c Department of Orthopedics, Rush University Medical Center, 1611 W Harrison St., Chicago, IL 60612, USA

**Keywords:** Adjacent segment disease, Cervical spondylosis, Cervical fusion, Cervical disc disorder, Postoperative complication, Revision surgery

## Abstract

**Background:**

Patients with long-term follow-up after cervical decompression and fusion have often been noted to have development of adjacent segment degeneration with a smaller subset of these patients progressing to adjacent segment disease (ASD), which results in the development of new symptomatic radiculopathy or myelopathy referable to a site either directly above or below a prior fused segment. The cause of ASD is multifactorial often involving natural age-related progression of spondylosis, accelerated progression following cervical decompression and fusion, operative technique, and patient-related factors. The effect of age at the time of index cervical decompression and fusion on the need for reoperation for ASD is not fully understood. This study aims to establish underlying risk factors for the development of symptomatic cervical ASD following cervical decompression and fusion requiring reoperation in patients of various age groups.

**Methods:**

A retrospective database review of patients aged 20 or greater with insurance claims of primary cervical decompression and fusion over the course of 11 years and 10 months (January 01, 2010–October 31, 2022) was conducted using an insurance claims database. The primary outcome was to evaluate the incidence of cervical ASD requiring reoperation amongst patients stratified by age at the time of their primary procedure. Secondary outcomes included an evaluation of various risk factors for ASD following cervical decompression and fusion including surgeon-controlled factors such as the number of levels fused and approach taken, patient cervical pathology including cervical disc disorder and cervical spondylosis, and underlying patient medical comorbidities including osteoporosis and vitamin D deficiency, and substance use.

**Results:**

A total of 60,292 patient records were analyzed, where the overall reoperation incidence for symptomatic ASD was 6.57%, peaking at 8.12% among those aged 30 to 39 and decreasing with age. Regression analysis revealed ages lower than 50 years as more predictive for the development of symptomatic ASD requiring reoperation. Multivariate regression analysis identified predictive factors for reoperation, including age, Elixhauser Comorbidity Index (ECI), multiple-level surgery, cervical spondylosis, cervical disc disorder, osteoporosis, and vitamin D deficiency. Notably, these factors had a variable impact across various age groups, as revealed by subgroup analysis.

**Conclusions:**

The incidence of reoperation secondary to symptomatic ASD is 6.57%, highest in those aged 30 to 39. The surgical approach had no significant impact on the need for reoperation, but multiple-level fusions posed a consistent risk in the development of symptomatic ASD requiring reoperation. Patient factors like degenerative disc disease, spondylosis, osteoporosis, and vitamin D deficiency were associated, urging further age-specific risk assessment and nonoperative intervention exploration.

## Introduction

Cervical spondylosis can result in cervical radiculopathy and myeloradiculopathy and may necessitate surgical intervention when nonoperative treatments fail or symptoms progress. Surgical management involves decompression of affected nerve roots or the spinal cord, typically through anterior or posterior approaches. Following decompression, stabilization is often achieved through a fusion procedure via the same approach. Cervical decompression and fusion have demonstrated significant clinical success in alleviating pain and enhancing neurologic symptoms, and are the standard of care for these conditions [1,2].

Patients with long-term follow-up after cervical decompression and fusion have often been noted to have development of adjacent segment degeneration with a smaller subset of these patients progressing to ASD, which results in the development of new symptomatic radiculopathy or myelopathy referable to a site either directly above or below a prior fused segment [3]. The cause of ASD is multifactorial involving natural age-related progression of spondylosis, accelerated progression following cervical decompression and fusion, operative technique, as well as patient-related factors [4]. Many studies have explored the relationship between the development of ASD and several factors, including the number and location of fusion segments, cervical spine sagittal alignment, preoperative range of motion, spinal canal stenosis, smoking history, and pre-existing degenerative changes at adjacent segments [4].

In current literature, the consensus on whether age plays and significant role in the reoperation rate for ASD is mixed [5], [6], [7], [8], [9]. Some studies claim there is no significant association between age and the need for reoperation for symptomatic ASD [5,6] while others found certain age groups to be predictive of symptomatic ASD requiring reoperation following cervical decompression and fusion [7], [8], [9]. In this study, we explore the role of natural aging and its impact on the development of symptomatic cervical ASD requiring surgical management. Additionally, we investigate how age influences the risk factors on the development of ASD that necessitates surgical intervention.

## Material and methods

### Data source

This study was conducted using the PearlDiver database (PearlDiver Technologies, IN, USA), specifically utilizing the Mariner161Ortho. This database covers a wide range of medical and prescription data, with records dating from January 01, 2010 to October 31, 2022 derived from provider networks. The database includes claims billed to all payer types, including commercial insurance, Medicare, Medicaid, self-pay, and more. The provided data were deidentified and compliant with the Health Insurance Portability and Accountability Act (HIPPA). The database is organized based on the diagnostic codes within the International Classification of Diseases-9 and International Classification of Diseases-10 classifications. We selected this database for analysis to ensure that a large population of patients was analyzed, thereby improving the power of the study, and reducing the risk of type II error.

### Eligibility criteria

The database was queried to identify patients who underwent primary cervical decompression and fusion and were initially screened to ascertain the absence of any prior history of cervical fusion. Subsequently, these patients were monitored for a duration of 365 days, and any patient records indicating a second cervical procedure within this period were excluded, ensuring the absence of reoperations. These records were further refined by excluding patients with any claims for opioid prescriptions during the 3 consecutive months leading up to the surgery. Additionally, patient records were monitored for a period of 10 years with the in-built “active” command within the PearlDiver database to ensure the absence of any deaths. Furthermore, records were filtered to exclude individuals below the age of 20 therefore all patients included were aged 20 years or older at the time of their primary procedure. All patients included in our study possessed a minimum of 10 years of postoperative follow-up data, commencing from the time of their index procedure. This implies that the patients included underwent their surgery between 2010 and 2012, allowing for a comprehensive 10-year follow-up period. Patients who did not require additional surgery were included in the control group and used as the reference population against which all comparisons were made.

### Study outcomes

A retrospective analysis of prospectively collected data over each patient's postoperative course was then performed. The primary outcome was the overall incidence of ASD requiring reoperation and incidence in each age cohort. Patients who underwent a procedure involving revision decompression and extension of fusion greater than 1 year after their index surgery were included and assumed to be a result of symptomatic ASD. Theoretically, only cases of severe refractory ASD were included as minor symptoms could have been resolved with conservative treatment such as physical therapy, injections, and medical management. This model, which uses a patient undergoing subsequent decompression and extension of fusion as a proxy to identify severe ASD requiring surgery, has also been testified and published for calculation of such incidences of ASD [10]. The true incidence of ASD after cervical decompression and fusion could be higher as not all patients require a return to the operating room. Our patient population was then stratified based on their age at the time of their index cervical decompression and fusion. Patient cohorts included the eighth decade and above (age 70 and greater), seventh decade (age 60–69), sixth decade (age 50–59), fifth decade (age 40–49), fourth decade (age 30–39), and third decade (age 20–29). Patients aged younger than 20 years were excluded from the analysis. Secondary outcomes included an evaluation of various risk factors including demographic factors (age, gender, Elixhauser Comorbidity Index, or ECI score), and 10 year continuous opioid use where the lax between first prescription and second is not more than 30 days, associated degenerative diagnosis (cervical spondylosis and cervical disc disorder) and medical comorbidities that may impact the bone health such as osteoarthritis, osteoporosis, vitamin D deficiency.

### Statistical analysis

The database was queried to assess the incidence of ASD in the patient population, as well as in the created age cohorts. Chi-square and T-test analyses were conducted to compare demographic characteristics, surgical approaches, surgical levels, medication abuse, comorbid conditions, and bone pathologies (see Appendix I). To ensure the linkage between the procedure and the respective approach codes, it was verified that the approach codes appeared on the same day as the procedure. Multiple-level codes were identified by filtering the population that had at least 1 associated multiple-level code.

A logistic regression was performed to determine whether any of the age groups were more or less predictive of developing ASD while controlling for all demographic factors that exhibited differences at baseline. The sixth age decade was used as the reference group, given that the average age of patients in the ASD group fell within that age range.

Additionally, all factors that were found to be significantly different at baseline were evaluated further through multivariate analysis. Variables that were found to be predictors of ASD development requiring reoperation were further assessed across each age group to determine if their impact was more pronounced in a particular age group using multivariate analysis.

Results are reported as adjusted odds ratios (OR), along with 95% confidence intervals and p-values. In the multivariate analysis, binary comparisons were employed for all discontinuous variables. This involved assessing the presence of the risk factor (True) compared with the absence of the risk factor (False). For instance, spondylosis was compared with not having spondylosis. We applied a Bonferroni correction to account for multiple comparisons, setting the threshold for statistical significance at a p-value of less than .02 and all values were reported as 2 significant figures. This adjustment helps reduce the risk of committing a type I error. All data were analyzed in aggregate form using R Statistical Software version 4.1.0 in the PearlDiver Software.

## Results

### Incidence

A total of 60,292 patient records were extracted, excluding reoperations within 1 year and patients who were opioid-naïve for the past 3 months. The overall incidence of reoperation secondary to ASD was 6.57% (3,692), with a range of 3.55% to 8.12% across different age groups (Table 1). Notably, the incidence peaked in the 30 to 39 years age group and subsequently decreased with advancing age. Logistic regression analysis, while adjusting for demographic, surgical, and comorbid predictors (including gender, ECI, surgical approach and levels, opioid use, cervical spondylosis and disc herniation, osteoporosis, and vitamin D deficiency), indicates that when using the fifth decade as the reference age group, the risk of developing ASD requiring reoperation following cervical fusion surgery increases in younger age groups (<50 years) and decreases in older age groups (>59 years) (Table 2).

**Table 1:** Incidence of adjacent segment disease (ASD) requiring reoperation in patients undergoing cervical fusion surgery: an age-stratified analysis.

Age cohort	Total (n = 60,292)	Percentage of patients in the age cohort (%)	ASD (n = 3,962)	Percentage of ASD in the age cohort (%)
Total	60,292			6.57
>69 y	4,932	8.20	175	3.55
60–69 y	13,590	22.56	728	5.36
50–59 y	20,971	34.83	1473	7.02
40–49 y	15,500	25.71	1187	7.66
30–39 y	4,633	7.69	376	8.12
20–29 y	603	1.00	22	3.65

**Table 2:** Logistic regression analysis of ASD incidence requiring reoperation: comparing age groups with the fifth decade as the reference category, while controlling for demographic, surgical, and comorbid predictors*.

Decade	OR	p-value
>69 y	0.37 [0.30, 0.46]	2.00^e-16^
60–69 y	0.58 [0.51, 0.66]	2.00^e-16^
50–59 y	Reference group	
40–49 y	1.42 [1.28, 1.58]	5.18^e-11^
30–39 y	1.96 [1.68, 2.28]	2.00^e-16^
20–29 y	0.59 [0.29, 1.07]	.11

⁎ Controlled predictors include- gender, ECI, surgical approach and levels, cervical spondylosis and disc herniation, osteoporosis, and vitamin D deficiency.

### Demographic characteristics

The demographic characteristics of patients who required reoperation secondary to ASD vs the control population are outlined in Table 3. Patients in the group requiring reoperation were notably younger (52.11 years vs. 54.96 years), had higher ECI scores (4.95 vs. 1.80), and were more likely to have undergone multilevel fusion procedures (48.51% vs. 43.29%). Furthermore, patients at risk of reoperation due to ASD had a significantly higher incidence of bone disorders such as cervical spondylosis (75.65% vs. 18.81%), cervical disc disorder (4.52% vs. 0.92%), osteoporosis (10.46% vs. 8.56%), and vitamin D deficiency (35.05% vs. 30.13%), (p-value < .02).

**Table 3:** Demographic factors, operative technique, and medical comorbidities amongst those with and without a diagnosis of symptomatic ASD requiring reoperation following cervical fusion surgery (2010–2022, n = 60,292).

	Control	Symptomatic ASD	p-value
	(n = 56,330)	(n = 3,962)	
Age (y)	53.96	52.11	**2.20^e-16^**
Gender			
*Man*	25,287 (44.89%)	1,723 (43.49%)	.09
*Woman*	31,043 (55.12)	2,239 (56.51%)	
ECI	1.80	4.95	**2.20^e-16^**
Approach			
*Anterior group*	51,334 (91.13%)	2,590 (65.37%)	**2.20^e-16^**
*Posterior group*	4,996 (8.87%)	82 (2.07%)	**2.20^e-16^**
Surgical Levels			
*Multiple*	24t,384 (43.29%)	1,922 (48.51%)	**1.65^e-10^**
*Single*	31,946 (56.71%)	2,040 (51.49%)	**1.65^e-10^**
Medication abuse			
*Continuous opioid use*	28 (0.05%)	*	1.00
Degenerative conditions			
*Spondylosis*	10,598 (18.81%)	2,037 (75.65%)	**2.20^e-16^**
*Disc disorder*	520 (0.92%)	167 (4.52%)	**2.20^e-16^**
Bone diseases			
*Osteoporosis*	4,823 (8.56%)	386 (10.46%)	**2.20^e-16^**
*Vitamin D deficiency*	16,970 (30.13%)	1,294 (35.05%)	**2.20^e-16^**

*PearlDiver does not report the number of records if the number falls below 11.

### Predictors of ASD requiring reoperation

Multivariable regression analysis revealed that age (aOR 0.96 [0.96, 0.97]), ECI (aOR 1.54 [1.52, 1.57]), multiple-level surgery (aOR 1.61 [1.47, 1.75]), cervical spondylosis (aOR 3.61 [3.31, 3.94]), and cervical disc disorder (aOR 2.45 [1.91, 3.13]) are independent risk factors that are predictive of developing symptomatic ASD requiring reoperation (Table 4). Age stratified analysis revealed that spondylosis and disc herniation pose an increased risk, across all age cohorts (Table 5). Having surgery at multiple levels becomes a stronger risk factor between the fourth and sixth decades.

**Table 4:** Multivariate regression analysis of the predictive factors requiring reoperation secondary to ASD.

Risk factor	aOR^+^	p-value
**Age (years)**	0.96 [0.96, 0.97]	**2.00^e-16^**
**ECI**	1.54 [1.52, 1.57]	**2.00^e-16^**
Approach		
*Anterior group*	8.06^E-12^ [2.41^E-170^, 2.69^E+147^]	.89
*Posterior group*	2.45^E-12^ [7.34^E-171^, 8.17^E+146^]	.89
Levels		
*Multiple group*	1.61 [1.47, 1.75]	**2.00^e-16^**
Degenerative conditions		
*Spondylosis*	3.61 [3.31, 3.94]	**2.00^e-16^**
*Disc disorder*	2.45 [1.91, 3.13]	**1.40^e-12^**
Bone disorders		
Osteoporosis	0.78 [0.66, 0.92]	**3.05^e-3^**
Vitamin D deficiency	0.66 [0.61, 0.73]	**2.00^e-16^**

**Table 5:** Multivariate analyses of factors predictive of developing ASD requiring reoperation: an age-stratified analysis.

Variables	Age cohorts					
	Second (20–29)	Third (30–39)	Fourth (40–49)	Fifth (50–59)	Sixth (60–69)	Seventh (>69)
ECI	0.80 [0.43,1.49]	0.99 [0.91, 1.07]	**1.06** **[1.01, 1.10]**	**1.04** **[1.01, 1.08]**	3.62 [1.33, 9.80]	1.03 [0.94, 1.12]
Surgical Levels (multiple)	2.68 [0.61, 11.79]	0.84 [0.65, 1.09]	**1.31** **[1.13, 1.51]**	**1.42** **[1.25, 1.63]**	**1.27** **[1.04, 1.56]**	1.20 [0.78, 1.83]
Spondylosis	**6.23** **[1.42, 27.31]**	**4.88** **[3.79, 6.28]**	**4.15** **[3.60, 4.79]**	**4.00** **[3.51, 4.57]**	**3.88** **[3.18, 4.72]**	**4.75** **[3.20, 7.05]**
Disc disorder	**33.70** **[3.91, 290.44]**	**2.71** **[1.39, 5.30]**	**2.82** **[1.86, 4.28]**	**2.69** **[1.89, 3.84]**	**4.54** **[2.79, 7.40]**	**3.62** **[1.33, 9.80]**
Osteoporosis	2.47 [0, ∞]	0.22 [0.03, 1.60]	0.70 [0.47, 1.06]	1.20 [0.96, 1.48]	1.01 [0.76, 1.33]	1.26 [0.77, 2.09]
Vitamin D deficiency	0.79 [0.13, 4.59]	1.10 [0.83, 1.46]	1.00 [0.86, 1.17]	0.93 [0.81, 1.08]	**0.80** **[0.64, 0.99]**	1.01 [0.66, 1.54]

Due to Bonferroni correction accounting for multiple comparisons, the threshold for statistical significance is at a p-value of less than 0.02.

## Discussion

Our study analyzed data from a significantly large patient population (n > 60,000) who underwent primary cervical decompression and fusion, a common treatment for cervical radiculopathy or myeloradiculopathy that is not responsive to nonoperative treatment.

The overall incidence of symptomatic ASD requiring reoperation after primary cervical decompression and fusion was 6.57%. Individuals in their fourth decade of life (ages 30–39) faced the highest risk, with an incidence rate of 8.12%. The overall incidence of symptomatic ASD in this study closely aligns with other large systematic reviews, which reported a pooled incidence ranging from 5.78% to 7.08% with younger males being at highest risk of developing ASD after anterior cervical discectomy and fusion (ACDF) [11,12]. Additionally, this study establishes a negative correlation between age and the risk of developing symptomatic ASD requiring reoperation, which is consistent with a systematic review suggesting that while older patients are more likely to develop cervical ASD, those more inclined to undergo reoperation for ASD are typically 60 years or younger [13]. Younger patients may be more prone to the development of ASD requiring reoperation due to more vigorous and prolonged use of their adjacent segments compared with older patients. Nevertheless, the analysis did reveal that age plays a role in the development of ASD requiring reoperation, particularly among younger age groups.

Patients who underwent multilevel fusion procedures had higher odds of requiring reoperation secondary to symptomatic ASD. Cervical fusion reduces the range of motion at the fusion site and increase mobility at adjacent levels, thereby increasing the mechanical load on these adjacent segments and elevating the risk of ASD [14]. The literature consistently reports that there is no significant difference in the risk of ASD between single and multiple-level surgeries [13].

This discrepancy could be attributed to the way ASD was defined in this study, specifically as cases requiring reoperation. Multilevel fusion procedures may increase the risk of reoperation due to ASD, but the overall risk of developing ASD may remain consistent. Goffin et al. [2] conducted long-term observations of ACDF patients and found that degenerative changes at the level adjacent to the fusion occurred in as many as 92% of patients. This supports the notion that a higher number of fused segments leads to greater motion restriction at the index level, creating a greater mechanical load at the adjacent segments. The slightly reduced association of reoperation due to ASD in older age groups, compared with younger age groups as shown in this study, may be explained by the common practice of adopting a more conservative approach in older patients to avoid unnecessary surgeries. Additionally, it could be attributed to patients being more willing to make lifestyle adjustments to reduce symptoms and, as a result, leading less active lives. Having a concurrent diagnosis of cervical disc disorder and spondylosis increases the likely of requiring reoperation for ASD. While this study, being a database study, cannot establish causality, this association appears logical since patients with these diagnoses are more likely to experience severe symptoms necessitating surgical intervention. In addition, the risk for reoperation for patients with cervical spondylosis increases linearly with the age given that spondylosis is a generative disorder [15]. To reduce the prevalence of symptomatic ASD in patients undergoing primary cervical decompression and fusion, there is growing consideration for cervical disc replacement (CDR) as a treatment option for cervical spondylosis and symptomatic cervical disc herniation [16]. While ACDF limits motion, leading to increased stress on adjacent segments, CDR is considered a motion-preserving procedure and may carry a lower risk of developing ASD. The reduced risk of ASD in patients undergoing CDR, compared with those who had ACDF, has been demonstrated in specific studies. However, it is essential to acknowledge that these trials exhibit a bias favoring CDR, with a substantial selection bias and, consequently, a lack of representation in ASD incidence. This lack of representation may also be related to cost reimbursement policies [17]. Other surgical risk factors associated with the development of symptomatic ASD after primary cervical decompression and fusion described previously in the literature and not evaluated within our study include ACDF plate positioning too close to the adjacent cervical disc, sagittal parameters such as high T1 slope, short segment fusions, and disruption of adjacent soft tissues [18].

Within this study, patient factors such as an underlying diagnosis of osteoporosis, vitamin D deficiency at the time of their index cervical decompression and fusion were at increased risk of developing symptomatic ASD requiring reoperation. While prior studies have demonstrated an increased risk of requiring adjacent level procedures in the lumbar spine for patients with a history of osteoporosis [18], and it was hypothesized that bone mineral density could affect the occurrence of degeneration of the neighboring segment this has yet to be established for the cervical spine and will require further evaluation. Similarly, various mechanisms for the development of ASD after cervical decompression and fusion have been proposed in the literature in patients with vitamin D deficiency, but no direct correlation has been shown [19,20].

The study's strengths include its large sample size, which increases the power of the study, and its long-term follow-up, which provides valuable information on the long-term risk of developing symptomatic ASD requiring revision cervical decompression and fusion. Furthermore, the comprehensive analysis adds to the existing literature on the risk factors associated with the need for reoperation after primary cervical decompression and fusion. However, the study has limitations as it relies on the accuracy and completeness of the diagnostic codes within the PearlDiver database. Additionally, confounding factors such as smoking history or preexisting degenerative changes at adjacent segments were not controlled for, which may limit the understanding of the complex relationship between age and subsequent development of symptomatic ASD. Finally, the study conducted an indirect estimation with all reoperations between 1 and 10 years after primary cervical decompression and fusion being treated as being a result of symptomatic ASD, which could include decompression and fusion of nonadjacent levels or reoperation for conditions other than ASD such as pseudoarthrosis or infection.

There are several potential avenues for future research based on the findings obtained during our study. First, a more in-depth exploration of the age-based risk of developing symptomatic ASD requiring reoperation after primary cervical decompression and fusion could be conducted, including controlling for potential confounding factors, such as smoking history, indication for the patient's index procedure, cervical plate positioning during an anterior based procedure or pre-existing degenerative changes at levels adjacent to those fused during the patient's primary procedure. Future studies could also explore the impact of nonoperative interventions, such as physical rehabilitation and medical management, on mitigating the risk of developing symptomatic ASD in older patients who undergo single or multilevel cervical decompression and fusion surgery. We anticipate that using the information obtained within this study, preoperative risk stratification and medical optimization can be improved to help reduce the risk of developing symptomatic ASD requiring reoperation after primary cervical decompression and fusion amongst patients of various age groups.

## Conclusion

In conclusion, our study of over 60,000 patients undergoing primary cervical decompression and fusion revealed that the overall incidence of symptomatic ASD requiring reoperation was 6.57%, with the highest risk observed in individuals aged 30 to 39 years. While the choice of surgical approach did not significantly influence the ultimate risk of developing symptomatic ASD requiring reoperation, multilevel fusions were predictive across fourth to sixth decade. Patient factors, including concurrent diagnosis of cervical spondylosis and cervical disc disorder as well as diseases affecting bone health such osteoporosis, and vitamin D deficiency were identified as potential risk factors. Future research should focus on refining age-based risk assessment and exploring nonoperative interventions to mitigate ASD risk in different age groups.

## Declaration of competing interest

The authors declare that they have no known competing financial interests or personal relationships that could have appeared to influence the work reported in this paper.

## Appendix Supplementary materials

Image, application 1
