# Supplementary material for: Exploring the incidence and risk factors of reoperation for symptomatic adjacent segment disease following cervical decompression and fusion
Source: N Am Spine Soc J. 2023 Dec 22;17:100305. doi: 10.1016/j.xnsj.2023.100305 (PMC10803933; doi:10.1016/j.xnsj.2023.100305)
Supplement: Supplementary file 1 [file mmc1.docx]

**Appendix 1**

| **Variable** | **Codes** |
| --- | --- |
|  |  |
| Cervical surgery | CPT-22548, CPT-22551, CPT-22585, CPT-22590, CPT-22595, CPT-22600 |
| Reoperation | CPT-22548, CPT-22551, CPT-22554, CPT-22552, CPT-22585, CPT-22590, CPT-22595, CPT-22600, CPT-22614 |
| Anterior approach | CPT-22548, CPT-22551, CPT-22585 |
| Posterior approach | CPT-22590, CPT-22595, CPT-22600 |
| Multiple levels | CPT-22585, CPT-22552, CPT-22614 |
| Opioids | USC-02211, USC-02212, USC-02214, USC-02221, USC-02222, USC-02231, USC-02232 |
| Cervical spondylosis | ICD-10-D-M47812, ICD-10-D-M4722, ICD-10-D-M4712, ICD-9-CM 721.1, ICD-9-CM 721.0, ICD-9-CM 721.0 |
| Cervical disc herniation | ICD-10-D-M5000, ICD-10-D-M501, ICD-10-D-M502, ICD-10-D-M503, ICD-10-D-M508, ICD-10-D-M509 |
| Osteoporosis | ICD-10-D-M810 |
| Vitamin D deficiency | ICD-10-D-E559, ICD-9-CM 268.9 |
|  |  |
|  |  |
